# Effect of microalgae on intestinal inflammation triggered by soybean meal and bacterial infection in zebrafish

**DOI:** 10.1371/journal.pone.0187696

**Published:** 2017-11-08

**Authors:** Karina Bravo-Tello, Nicole Ehrenfeld, Camila J. Solís, Pilar E. Ulloa, Manuel Hedrera, Marjorie Pizarro-Guajardo, Daniel Paredes-Sabja, Carmen G. Feijóo

**Affiliations:** 1 Departamento de Ciencias Biologicas, Facultad de Ciencias Biologicas, Universidad Andres Bello, Santiago, Chile; 2 Interdisciplinary Center for Aquaculture Research, Concepción, Chile; 3 Centro de Investigación Austral Biotech, Escuela de Biotecnología, Universidad Santo Tomás, Santiago, Chile; 4 Facultad de Medicina Veterinaria y Agronomia, Escuela de Agronomia, Universidad de Las Américas, Santiago, Chile; 5 Microbiota-Host Interactions and Clostridia Research Group, Departamento de Ciencias Biológicas, Facultad de Ciencias Biológicas, Universidad Andrés Bello, Santiago, Chile; Universitat Politècnica de València, SPAIN

## Abstract

Soybean meal has been used in many commercial diets for farm fish; despite this component inducing intestinal inflammation. On the other hand, microalgae have increasingly been used as dietary supplements in fish feed. Nevertheless, the vast quantity of microalgae species means that many remain under- or unstudied, thus limiting wide scale commercial application. In this work, we evaluated the effects to zebrafish (*Danio rerio*) of including *Tetraselmis sp* (Ts); *Phaeodactylum tricornutum* (Pt); *Chlorella sp* (Ch); *Nannochloropsis oculata* (No); or *Nannochloropsis gaditana* (Ng) as additives in a soybean meal-based diet on intestinal inflammation and survival after *Edwardsiella tarda* infection. In larvae fed a soybean meal diet supplemented with Ts, Pt, Ch, or Ng, the quantity of neutrophils present in the intestine drastically decreased as compared to larvae fed only the soybean meal diet. Likewise, Ts or Ch supplements in soybean meal or fishmeal increased zebrafish survival by more than 20% after being challenged. In the case of Ts, the observed effect correlated with an increased number of neutrophils present at the infection site. These results suggest that the inclusion of Ts or Ch in fish diets could allow the use of SBM and at the same time improve performance against pathogen.

## Introduction

Quality nutrition is of primary importance in fish farming as this aspect can dictate the health status and growth of fish, two issues directly linked to aquaculture viability. Recent decades have witnessed increased worldwide demand for fishmeal (FM) and fish oil, leading to consequently increased costs and lower availability for these products [1]. This situation has driven the industry to search for environmentally-friendly, sustainable alternatives. In particular, plant protein sources are being evaluated as a FM replacement [2]. Among plant protein sources, research focus has been given to cereals and legumes, with soybean meal (SBM) so far being the most used due to high availability, low and stable costs over the last 30 years, high digestible protein contents, and a balanced amino acid profile [3].

However, SBM has anti-nutritional factors that generate different degrees of intestinal inflammation in both carnivorous (e.g. Atlantic salmon [*Salmo salar* L.] and rainbow trout [*Oncorhynchus mykiss*]) and omnivorous fish (e.g. carp [*Cyprinus carpio* L.] and zebrafish [*Danio rerio*]) [4–8]. This pathology is a detrimental condition that interferes with nutrient digestion, absorption, and/or utilization, affecting growth and health with consequences in the ability to respond to pathogens [9]. Therefore, a key challenge facing SBM use in fish diets is doing so while also preventing or decreasing negative, pathological effects, such as inflammation. One possible solution currently being tested in fish is the incorporation of additives that offer intestinal protection against inflammation [9,10].

For example, mannan-oligosaccharide supplements decrease the detrimental effects of intestinal inflammation in Atlantic salmon [9]. Furthermore, a recent study by Ulloa *et al*. [10] demonstrated that the lactoferrin supplements prevent SBM-induced intestinal inflammation in zebrafish while also improving performance against bacterial infection with *Edwardsiella tarda*. Therefore, the supplementation of SBM-based diets with additives appears a plausible alternative to cope with intestinal inflammation. Beyond this, additives could exert additional functions, such as in the response to pathogenic infections.

In working towards sustainable aquaculture practices, marine microalgae have been increasingly incorporated in aquafeeds as fish oil alternatives or to improve the immune response of fish [11–13]. Microalgae are a diverse group of microorganisms, including diatoms, dinoflagellates, and flagellates, and contain up to 70% protein, 15–30% carbohydrates, 30–50% lipids (as glycerol and omega 3 and 6 fatty acids), up to 14% carotene, and a high diversity of vitamins (e.g. A, B1, B2, B6, B12, C, E, biotin, folic acid, and pantothenic acid) [14]. Several studies have evaluated the beneficial effect of microalgae in farmed fish.

For example, a study in gilthead sea bream (*Sparus aurata*) showed that *Ulva lactuca* and *Pterocladia capillacea* improved growth performance, feed utilization, nutrient retention, and survival to hypoxia stress [15]. Later, Cerezuela *et al*. [16] demonstrated that *Tetraselmis chuii* and *Phaeodactylum tricornutum* (Pt) increase sea bream survival to pathogenic challenges, in addition to showing a significant influence of these microalgae on the intestinal morphology and microbiota [17]. Similarly, *Chlorella* sp. modulated adaptive and innate immunities in Gibel carp (*Carassius Auratus Gibelio*) by increasing liver and kidney levels of the M and D immunoglobulins, interleukin-22, and chemokine CC5 [18]. Furthermore, one study demonstrated that *Chlorella vulgaris* decreases the intestinal inflammation triggered by SBM in Atlantic Salmon [19]. Due to the broad spectrum of beneficial effects exerted by microalgae, we decided to analyze if microalgae could simultaneously control two of the major problems affecting the aquaculture industry—intestinal inflammation and pathogen infection.

Overall, evaluating many diets supplemented by different microalgae directly in aquaculture species is a high cost and long-term endeavor. Consequently, new strategies are needed to accelerate experimental dietary supplement processing and to make this task cost-effective [20]. One plausible strategy is to perform preliminary studies in zebrafish, an animal model in which many diets can be assessed short-term and at lowers costs than at fish farms [8]; [20][10]. A particular advantage of zebrafish is the availability of transgenic lines such as Tg(BACmpo:GFP)^i114^, a line in which innate immune cells (i.e. neutrophils) are fluorescently labeled [21]. Since neutrophil migration is a key step during intestinal inflammation, these cells can be used as inflammatory markers and monitored *in vivo* in the whole organism. This strategy has been used before by Hedrera *et al*. (Hedrera *et al*. 2013), who demonstrated that soybean meal consumption by zebrafish results in inflammatory side effects similar to those observed in commercially farmed fish. A primary advantage of using transgenic, fluorescently-labeled zebrafish is that the inflammatory process can be very quickly observed, even before histological effects become recognizable.

In this work, we evaluated the effect of including different microalgae as additives to a SBM-based diet (*Tetraselmis* sp [Ts].; Pt; *Chlorella* sp. [Ch]; *Nannochloropsis oculata* [No], and *Nannochloropsis gaditana* [Ng]), with considerations given to intestinal inflammation and the immune response against *E*. *tarda* infection. Among the results, Ts drastically decreased intestinal inflammation and improved performance against *Edwarsiella tarda*.

## Results

### Effects of microalgae on intestinal inflammation

**Fig 1:**
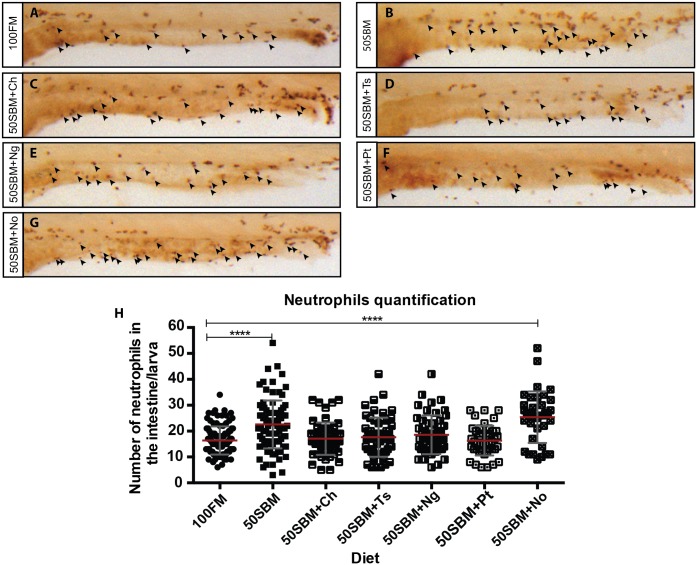
Effect of microalgae on intestinal inflammation. (A-G) Lateral view of 9 dpf Tg(BACmpo:GFP)^i114^ larvae after four days of feeding with the different diets; fishmeal (100FM), soybean meal (50SBM), and soybean meal + microalgae: 50SBM+Ch, 50SBM+Ts, 50SBM+A3Ng, 50SBM+Pt, or 50SBM+No. Black arrowheads indicate neutrophils. (H) The amount of intestinal neutrophils was quantified by immunohistochemistry against GFP. At least 25 larvae per diet were analyzed in each three different experiments. Statistical analysis was conducted using a non-parametric one-way ANOVA. ***P < 0.0001. Red bars represent the mean, and gray bars represent standard deviation.

To determine if microalgae protect against SBM-induced intestinal inflammation, five different microalgae species were added to fishmeal (100FM) or soybean meal (50SBM) diets, thus generating ten experimental diets (100FM+Ch, 100FM+Ng, 100FM+No, 100FM+Pt, 100FM+Ts, 50SBM+Ch, 50SBM+Ng, 50SBM+No, 50SBM+Pt, 50SBM+Ts). Media containing 50SBM and 100FM without any additives were used as positive and negative controls, respectively. To determine the existence of intestinal inflammation, the amount of neutrophils present in the intestine was used as an inflammatory marker, thus neutrophils were quantified after four days of feeding with the experimental diets (Fig 1).

As expected, and as previously described [8], larvae fed with 50SBM showed a clear increase in intestinal neutrophil quantities as compared to larvae fed with 100FM (Fig 1A, 1B and 1H). Larvae fed the 50SBM diet supplemented with Ch, Ts, Ng, or Pt had fewer intestinal neutrophils as compared to larvae fed with 50SBM alone. Moreover, the intestinal neutrophil quantities of these experimental larvae (i.e. 50SBM+Ch, Ts, Ng, or Pt) were similar to larvae fed with 100FM alone (Fig 1A, 1C–1F and 1H). In contrast, larvae fed with 50SBM+No had similar neutrophil amounts in the intestine as larvae fed with 50SBM alone (Fig 1B, 1G and 1H). Interestingly, larvae fed the supplemented 100FM diets showed no significant differences in intestinal neutrophil quantities as compared to larvae fed with 100FM alone (S1 Fig).

### Effect of microalgae on fish response to Edwardsiella tarda infection

**Fig 2:**
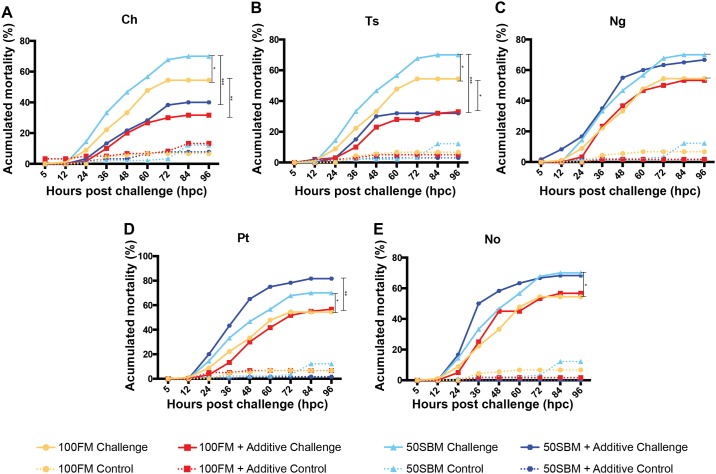
Effect of microalgae on fish response to *Edwarsiella tarda* infection. Tg(BACmpo:GFP)^i114^ larvae fed with experimental or control diets were challenged with *E*. *tarda* for 5 h. Mortality was monitored every 12 h until 96 hour post challenge (hpc). Unchallenged larvae were subjected to the same feeding strategy and monitored during the same period. The graph represents the result obtained from three independent experiments. Statistical analysis was performed using survival curve analysis with Log-rank test against the 100FM and 50SBM diets. *P < 0.05, **P < 0.01, ***P < 0.001. Solid lines represent challenged larvae and dotted lines represent control (unchallenged) larvae.

To determine the effects of microalgae on host performance against pathogens, larvae fed with the experimental diets were challenged with the bacterium *E*. *tarda* (Fig 2). Larvae fed with 50SBM had significantly higher mortality rates than larvae fed with 100FM (P < 0.05) (Fig 2). The addition of Ch to 50SBM and 100FM decreased larvae mortality by 30% and 23%, respectively, as compared to the control groups (Fig 2A). The addition of Ts to 50SBM and 100FM also produced drastic effects, decreasing larvae mortality by 38% and 21%, respectively, as compared to the control groups (Fig 2B). Both the Ch and Ts supplemented diets produced similar larvae mortality rates, with no significant differences (Fig 2A and 2B). On the other hand, the addition of Ng, Pt, or No to 50SBM and 100FM did not have any positive effects on larval performance against *E*. *tarda* (Fig 2C–2E).

### Microalgae improve immune performance against bacteria

**Fig 3:**
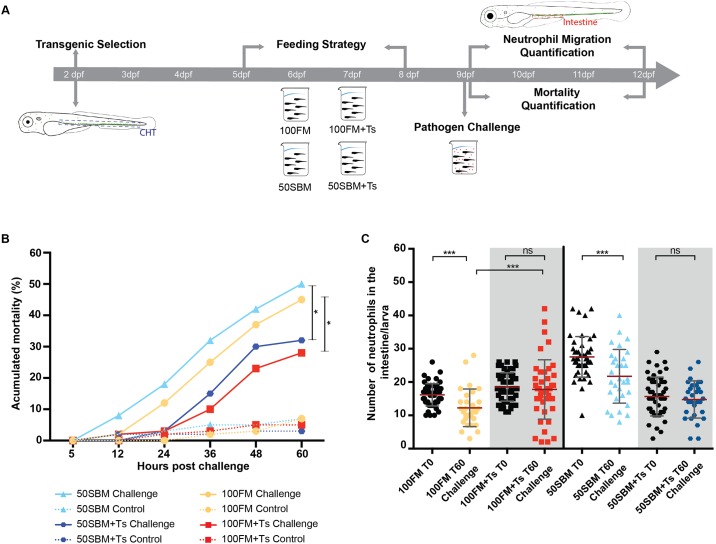
Microalgae improve neutrophil behavior after infection. (A) Assay strategy. (B) Accumulated mortality in challenged (continuous line) and unchallenged (doted line) Tg(BACmpo:GFP)^i114^ larvae fed with 100FM or 50SBM with and without *Tetraselmis* sp. (Ts). Mortality was monitored every 12 h until 60 hours post challenge (hpc). (C) Amount of intestinal neutrophils before challenge (T0), and at 60 hpc (T60 *Challenge*). At least 25 larvae per condition were analyzed in each three independent experiments. Statistical analysis was conducted using a non-parametric Mann-Whitney test. *P < 0.5, ***P < 0.001. Red bars represent the mean, and gray bars represent standard deviation.

*Tetraselmis* sp was one of the two microalgae that most protected larvae against *E*. *tarda* infection. Given that there is no information about Ts effects on the innate immune response, a subsequent assay was performed to determine if decreased mortality was directly related to an improved innate immune response. Since it is reported that *E*. *tarda* induce a systemic infection, colonizing several organs [22,23], we decided to focus at the intestine because we confirm their presence on it (S2 Fig). Thus, again neutrophils were used as inflammatory markers and were quantified in this organ before challenge (T0) and after 60 hours post challenge (hpc) in larvae fed wit control diets and supplemented with additives. Also, mortality was monitored in challenge and unchallenged larvae fed with control and experimental diets (Fig 3A). After 60 hpc larvae fed with Ts-supplemented diets (both 100FM+Ts and 50SBM+Ts) had significantly less mortality as compared to larvae fed with control diets (Fig 3B). On the other hand and regarding neutrophils amount present in the intestine, larvae fed with 100FM or 50SBM had significantly fewer neutrophils at the intestine after 60 hpc when compared to their corresponding T0. On the contrary, after 60hpc larvae fed with 100FM+Ts and 50SBM+Ts had similar amount of intestinal neutrophils compared to T0 (Fig 3C). It is important to remember that we already demonstrated that Ts avoid intestinal inflammation (Fig 1D and 1H), thus the amount of intestinal neutrophils present at T0 in larvae fed with 50SBM+Ts is considerably lower then that found in larvae fed with 50SBM. Finally after 60 hpc larvae fed with 100FM+Ts have more neutrophils at the intestine then those fed with 100FM (Fig 3C).

## Discussion

In recent decades, different microalgae species, alone or mixed, have been tested for aquaculture applications, with variable results [11,24,25]. Although microalgae can inherently vary in nutritional value, a trait possibly further affected under culture conditions, microalgae contain high levels of omega-3 fatty acids, bioactive cell wall compounds such as β-glucans, and a multitude of other bioactive components, such as β-carotenes, flavonoids, nucleotides, and water-soluble peptides [26,27]. All of these factors could modulate fish physiology, thereby promoting general well-being and better overall health.

This study analyzed the effects of five microalgae, including *Tetraselmis* sp., *P*. *tricornutum*, *Chlorella* sp., *N*. *oculata*, and *N*. *gaditana* on a SBM-triggered intestinal inflammation and infection-induced mortality in zebrafish larvae. While all microalgae, except No, decreased the intestinal inflammation triggered by a SBM diet, Ts and Ch also decreased larval mortality after infection with *E*. *tarda*. Additionally, it was determined that the ability of Ts to reduce mortality was correlated with an increased quantity of neutrophils at the site of infection.

The protective effect of microalgae against SBM seemed generalized as four of the five tested microalgae effectively granted protection. Moreover, it seems that microalgae prevent intestinal inflammation owing to the amount of neutrophils present in the intestine of those larvae fed with SBM+microalgae (except No) is indistinguishable from that observed in larvae fed with FM. This is in line with other reports, where microalgae supplementation (e.g. with *Scizochytrium* sp and *Navicula* sp) increases the mucus production and globet cell number in the intestines of Atlantic salmon and Pacific red snapper (*Lutjanus peru*) [28,29]. Since mucus is an important physical barrier that protects the intestine from different stressors, increased mucus could help to prevent the harmful effects of SBM.

The exact mechanism by which SBM produces intestinal inflammation is unknown. The available evidence suggests that this legume disrupts intestinal barrier integrity by increasing permeability and by partially disturbing intestinal cell-cell tight junction components, such as ocludins and claudins [30,31]. Related to this, dietary supplementation with *Navicula* sp, *T*. *chuii*, or Pt significantly increases the transcriptional levels of ocludins in gilthead seabream [13,32], supporting the hypothesis that microalgae could prevent SBM detrimental effect by favoring the maintenance of intestinal epithelial barrier integrity. Thus, in the current study, the intestinal protective effects of microalgae against SBM could be dual–(1) by increasing the intestinal mucus layer and (2) by increasing cell-cell union resistance.

Whereas nearly all assessed microalgae exerted protective intestinal effects, only two microalgae, Ts and Ch, evidenced beneficial effects in decreasing larval mortality to bacterial infection. Available research indicates that *Tetraselmis suecica* exerts anti-bacterial activities against different *Vibrio* strains *in vitro* [33], while *C*. *vulgaris* confers protection against *Escherichia coli* and *Listeria monocytogenes* infection in mice [34,35]. The mechanism by which *C*. *vulgaris* enhances bacterial resistance is mediated by humoral and cellular processes [34–37]. Specifically, pro-inflammatory cytokine (e.g. IL-1α, MIP, and TNFα) levels rise, enhancing neutrophils migration to the infection site. In the present study, we showed that after 60 hours post challenge, neutrophils amounts in the intestine decrease compared to that observed before challenge, independent of the diet (100FM or 50SBM) administered. On the contrary, when diets were supplemented with Ts the number of neutrophil remained indistinguishable before and after challenge. In addition, Ts decreased larval mortality after bacterial challenge. Thus, considering our results and the previous antecedents mentioned above, we hypothesize that Ts could modulate immune response by improving either neutrophils migration or enhancing the number of neutrophils that migrate to the infected site. A higher amount of neutrophils would fight the infection more efficiently, thereby enhancing larval survival. The fact that the amount of neutrophil found at T60 in larvae fed with 100FM+Ts is higher than that observed in larvae fed with 100FM alone support our hypothesis.

The gastrointestinal tract is chronically exposed to a vast number of dietary antigens, microorganisms, and toxic molecules. Therefore, the ability of the intestine to protect against potentially harmful molecules and pathogenic bacteria is crucial in maintaining general homeostasis. Indeed, intestinal epithelial cells are the first contact point with the external environment, acting as the first line of host defense against potentially harmful agents. Maintaining the stability and integrity of the intestinal epithelium is critical for proper nutrition, defense, and growth in farmed fish. Regarding this, microalgae dietary supplements could not only improve host defenses against pathogens, thus decreasing antibiotic use, but also pave the way for more sustainable and environmentally-friendly fish diets by facilitating the partial replacement of fishmeal with soybean meal.

## Materials and methods

### Microalgae strains and culture conditions

*Nannochloropsis gaditana* (Ng) and *Tetraselmis* sp. (Ts) samples were provided by the Desert Bioenergy Consortium, while *Nannochloropsis oculata* (No), *Chlorella* sp. (Ch), and *Phaeodactylum tricornutum* (Pt) samples were provided by the Aeon Biogroup SpA. All strains were supplied as a dry powder. Microalgae were cultured in open ponds filled with the F/2 medium [38] diluted in either seawater (for No, Ng, Ts, and PT) or freshwater (for Ch). Pond temperatures ranged from 10°C to 25°C. After reaching the late exponential growth phase, microalgal cultures were diluted daily at a rate corresponding to 50% of the maximum growth rate. Cultures were then centrifuged and spray-dried, excepting Ts, which was dried using a solar oven.

### Diet preparation

Seven diets were prepared as indicated in Tables 1 and 2. A SBM diet (50SBM) was used as a positive control, and a FM diet (100FM) was used as a negative control. Experimental diets were prepared by separately supplementing the 50SBM or 100FM diets with each of the microalgae species at a concentration of 10 g kg^-1^ (i.e. 100FM+Ch, 100FM+Ng, 100FM+No, 100FM+Pt, 100FM+Ts, 50SBM+Ch, 50SBM+Ng, 50SBM+No, 50SBM+Pt, and 50SBM+Ts). All diets were formulated and manufactured by extrusion at the Animal Feed Pilot Plant of the Aquaculture School, Universidad Católica de Temuco, Chile, as previously described [10]. Briefly, lyophilized microalgae were dissolved in fish oil and incorporated into diets using a laboratory vacuum coater (Dinnissen model VC10, Sevenum, Netherlands). Diets were formulated to be isoenergetic and isonitrogenous, and were supplemented with a vitamineral premix. Proximal composition analyses of the diet were performed at Universidad Católica de Temuco, Chile according to the following procedures: dry matter was obtained after 24 h in an oven at 105°C; ash by combustion at 450°C for 16 h, protein (N • 6.25) by the Kjeldahl method; fat by the Soxhlet method; and gross energy by caloric factor (4, 9, and 4 for protein, lipid, and carbohydrate respectively).

**Table 1:** Ingredients and nutrient composition of control and experimental diets.

Ingredients g kg^-1^	Fish meal (100FM)	Soybean meal (50SBM)
Fishmeal	555	250
Soybean meal	0	500
Wheat grain meal	255	110
Starch	60	60
Fish oil	30	60
Vitamineral mix^1^	20	20
Cellulose	80	0
*Chlorella sp (Ch)*	--	--
*Nannochloropsis gaditana (Ng)*	--	--
*Phaeodactylum tricornutum (Pt)*	--	--
*Nannochloropsis oculata (No)*	--	
**Total**	1000	1000
**Analytical composition (dry bases, %)**		
Dry matter	94.42 (±0.13)	93.10 (±0.17)
Crude protein	43.76 (±0.58)	45.44(±2.30)
Crude lipids	6.54 (±0.25)	7.05(±0.24)
Ash	9.730 (±0.07)	8.38 (±0.12
Gross energy (MJ kg^-1^)	20.0 (±0.06)	20.2 (±0.06)

^1^As recommended by the NRC (1993).

**Table 2:** Control and experimental diets.

Microalgae	Fish meal (100 FM)	Soybean meal (50 SBM)	Microalgae supplementation (g kg ^-1^)
Chlorella sp (Ch)	100FM + Ch	50SBM + Ch	10
Tetraselmis sp (Ts)	100FM + Ts	50SBM + Ts	10
Nannochloropsis gaditana (Ng)	100FM + Ng	50SBM + Ng	10
Phaeodactylum tricornutum (Pt)	100FM + Pt	50SBM + Pt	10
Nannochloropsis oculata (No)	100FM + No	50SBM + No	10

### Zebrafish strain and maintenance

The transgenic zebrafish line Tg(BACmpo:GFP)^i114^ was used in all experiments [21]. Zebrafish were maintained and raised at the Fish Immunology Laboratory, Universidad Andrés Bello according to standard protocols [39]. All embryos were collected by natural spawning, staged according to Kimmel *et al*. [40], and raised in Petri dishes at 28.5°C in the E3 medium (5 mM NaCl, 0.17 mM KCl, 0.33 mM CaCl_2_, 0.33 mM MgSO_4_, methylene blue, pH 7.0) as previously described [41]. Embryonic and larval stages were expressed as hours post-fertilization (hpf) or days post-fertilization (dpf). All animal handling procedures were approved by the "Committee of Animal Bioethics of the Universidad Andres Bello". Certificate number 025/2013. During feeding assay larvae do not suffer any pain and that survive without any problem, thus, we do not euthanized them. For the bacterial challenge assay, we euthanized the larvae that survive using an overdose of 4% Tricaine (Sigma).

### Experimental feeding

Experimental feeding was conducted as previously described by Hedrera *et al*. [8]. Briefly, 30 larvae in 100 ml of aquarium water were fed with a specific diet (Tables 1 and 2) twice daily from 5 to 8 dpf, with intervals of at least 6 h between feedings. Aquarium water was replaced ones a day. Larvae were fixed 19 h after the last meal to promote intestinal emptying (Fig 3A). Each feeding assay was performed in triplicate and at least 3 times.

### Immunohistochemistry and neutrophil quantification

Immunohistochemistry was performed as previously described [42]. Briefly, larvae were fixed for 1 h in 4% paraformaldehyde/phosphate buffered saline, dehydrated in 100% methanol, and stored at -20°C until processing. The antibodies used were rabbit anti-GFP (Life Technologies, Cat N° A11122) and anti-rabbit peroxidase (Sigma, Cat N° A8275). Neutrophils infiltrating the intestine were quantified at the mid and posterior sections, as described by Hedrera *et al*. [8]. A minimum of 25 larvae was analyzed per condition in three independent experiments.

### *Edwardsiella tarda* transformation and challenge

*Edwardsiella tarda* FL60 was kindly provided by Dr. Phillip Klesius (USDA, Agricultural Research Service, Aquatic Animal Health Research Unit). Transformation of *E*. *tarda* was attempted by several ways (conjugation with HB101 E. coli and transformation in quimiocompetent cells) which were unsuccessful. It was only possible to obtain transformed *E*. *tarda* cells by electroporation. Electro-competent cells of *E*. *tarda* were prepared, growing *E*. *tarda* in SOB (2% tryptone, 0,5% yeast extract, 10 mM NaCl, 2,5 mM KCl) until OD600 0.5. After 2 washes with water, cells were store in 10% glycerol at -80°C until use. Transformation with plasmid pMP7604, which carries mCherry under the control of tac promoter and tet resistance, was made by electroporation. Then, it was seeded in plates with tetracycline (10 ug/ml) and grow at 28°C until colonies appear. Clones were check for luminescence. The *E*. *tarda* challenge was performed as previously described by Ulloa *et al*. [10]. Briefly, *E*.*tarda* was grown overnight at 28°C in trypticase soy broth (TSB). The overnight culture was diluted to 1:100 in fresh TSB at 28°C until reached 10^8^ CFU/mL. The culture was pelleted and washed as previously described and then suspended to reach 10^8^ CFU/mL in aquarium water (*E*. *tarda* water medium). After 4 days of feeding, a group of 30 larvae were challenged for 5 h in 200 mL of *E*. *tarda* water medium and then changed to a tank with new aquarium water. Each respective diet was resumed for the remainder of the trial period, and larvae mortality was monitored every 12 h for 4 days. As a challenge assay negative control, larvae were maintained in aquarium water following the same feeding strategy as challenged larvae. Mortality was monitored in parallel to challenged larvae. This assay was performed at least in three independent times.

### Statistical analysis

Neutrophil quantification data were analyzed using the non-parametric Kruskal-Wallis test and one-way ANOVA. Survival data were analyzed using the Kaplan-Meier method, and group differences were analyzed by the Log-rank test, using the Bonferroni correction for multiple comparisons. All analyses were performed using Prism 6 (GraphPad Software, La Jolla, CA, USA). Differences were considered statistically significant when at p< 0.05.

### Imaging

Larvae images were taken using a QImaging MicroPublisher 5.0 RVT camera attached to an Olympus SZX16 stereoscope. Confocal images were acquired with Olympus FluoView FV1000 Spectral Confocal Microscope (software version 2.1). All images were processed with Photoshop CS6 and ImageJ 1.44o, showing the representative effects of each treatment.

## Supporting information

S1 Fig Effect of microalgae on immune response. (A-G) Lateral view of 9 dpf Tg(BACmpo:GFP)^i114^ larvae after four days of feeding with the different diets; fishmeal (100FM), soybean meal (50SBM), and fishmeal + microalgae: 100FM+Ch, 100FM+Ts, 100FM+A3Ng, 100FM+Pt, or 100FM+No. Black arrowheads indicate neutrophils. (H) The amount of intestinal neutrophils was quantified by immunohistochemistry against GFP. At least 25 larvae per diet were analyzed in three different experiments. Statistical analysis was conducted using a non-parametric one-way ANOVA. ****P < 0.0001. Red bars represent the mean, and gray bars represent standard deviation.
(TIF)

S2 Fig Intestinal localization of *Edwardsiella tarda* after challenge assay. (A, D). Lateral view of a mid-intestine section from a 12dpf larva. (B, E) mCherry labeled *Edwardsiella tarda*. (C, F) Merge of both images showing the presence or not of *Edwardsiella tarda* in the intestine.
(TIF)

## Abbreviations

Ch: *Chlorella* sp
dpf: days post-fertilization
FM: fishmeal
hpc: hours post-challenge
hpf: hours post-fertilization
Ng: *Nannochloropsis gaditana*
No: *Nannochloropsis oculata*
Pt: *Phaeodactylum tricornutum*
SBM: soybean meal
Ts: *Tetraselmis* sp.
