# Supplementary figures and images for: Effect of microalgae on intestinal inflammation triggered by soybean meal and bacterial infection in zebrafish
Source: PLoS One. 2017 Nov 8;12(11):e0187696. doi: 10.1371/journal.pone.0187696 (PMC5678869; doi:10.1371/journal.pone.0187696)

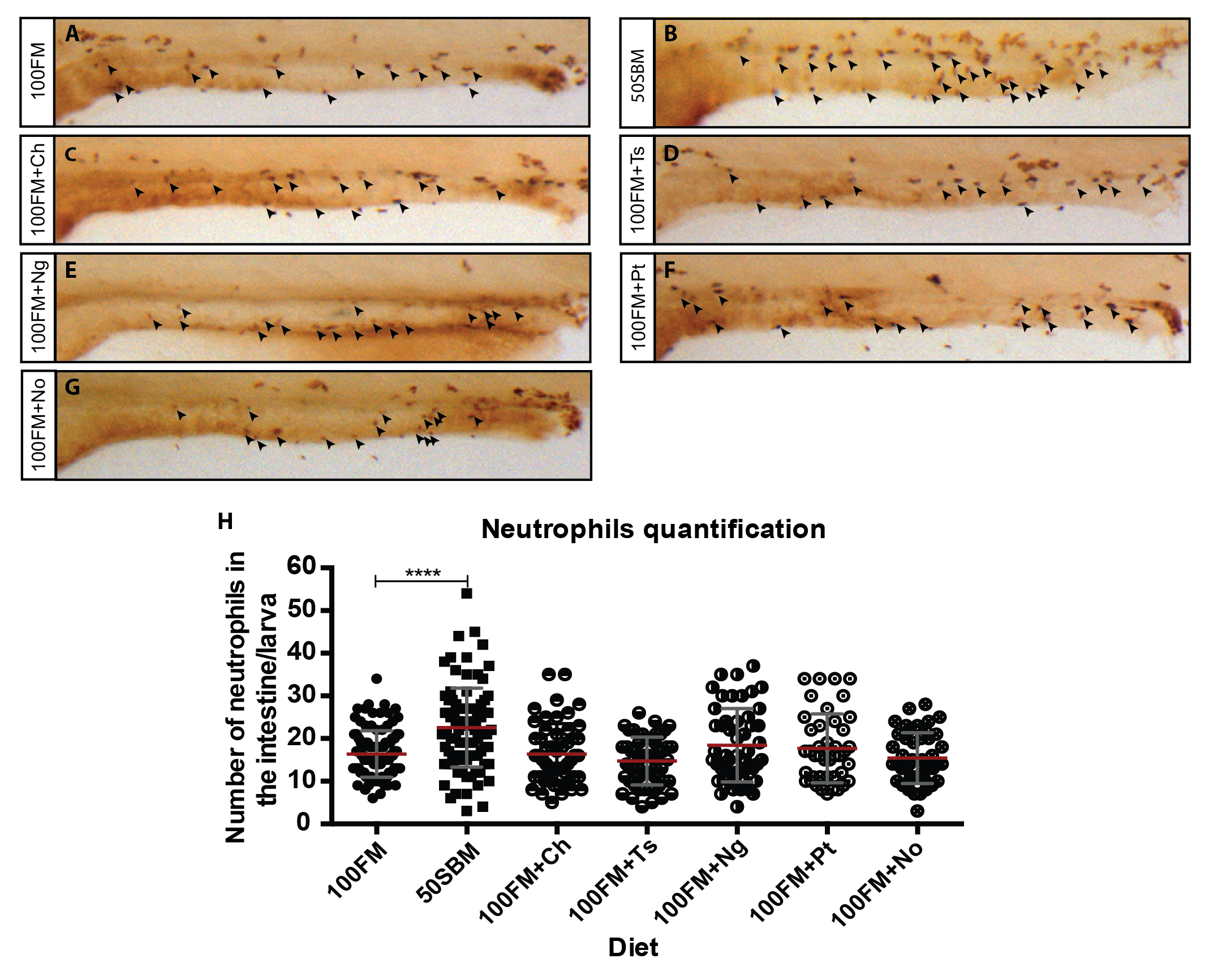

Supplement: S1 Fig — (A-G) Lateral view of 9 dpf Tg(BACmpo:GFP)i114 larvae after four days of feeding with the different diets; fishmeal (100FM), soybean meal (50SBM), and fishmeal + microalgae: 100FM+Ch, 100FM+Ts, 100FM+A3Ng, 100FM+Pt, or 100FM+No. Black arrowheads indicate neutrophils. (H) The amount of intestinal neutrophils was quantified by immunohistochemistry against GFP. At least 25 larvae per diet were analyzed in three different experiments. Statistical analysis was conducted using a non-parametric one-way ANOVA. ****P < 0.0001. Red bars represent the mean, and gray bars represent standard deviation. (TIF) [file pone.0187696.s001.tif]

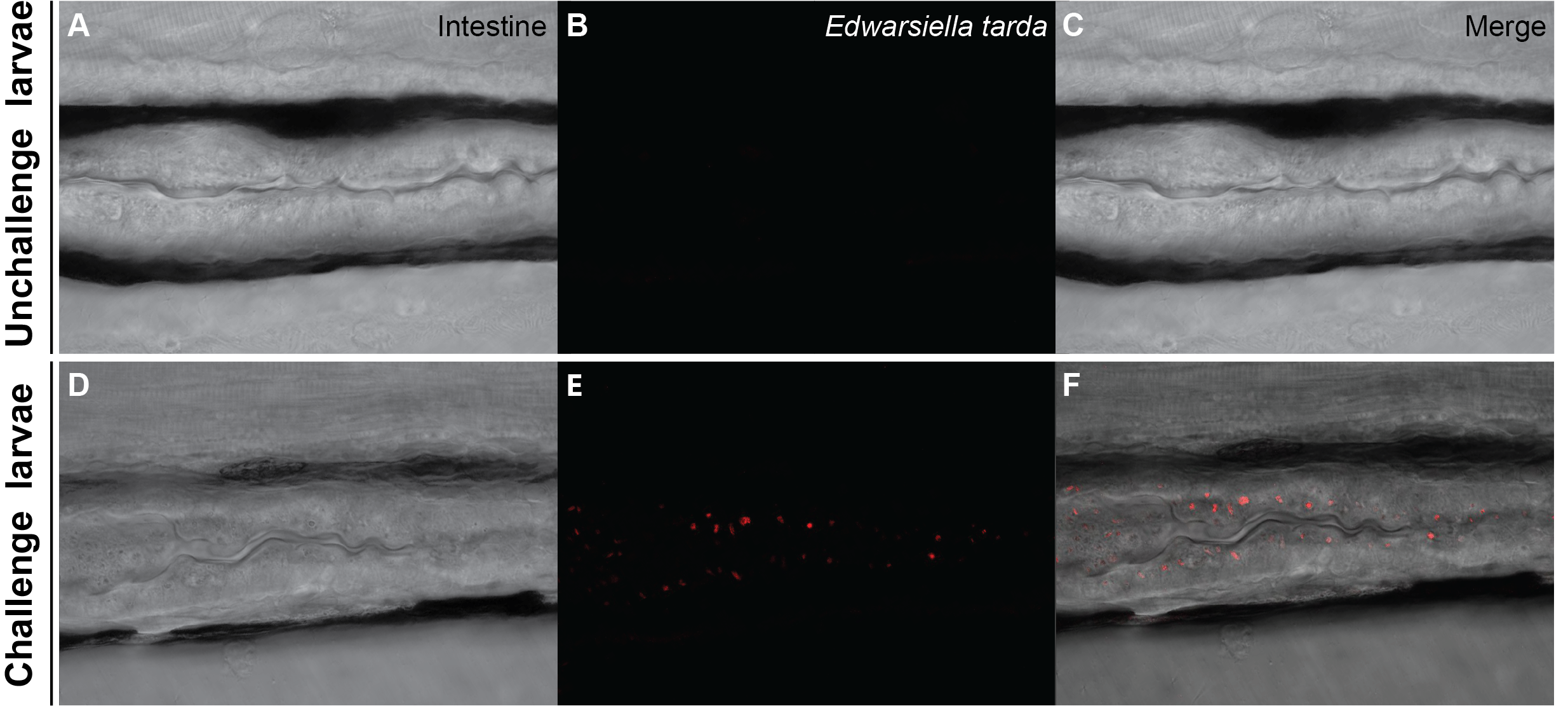

Supplement: S2 Fig — (A, D). Lateral view of a mid-intestine section from a 12dpf larva. (B, E) mCherry labeled Edwardsiella tarda. (C, F) Merge of both images showing the presence or not of Edwardsiella tarda in the intestine. (TIF) [file pone.0187696.s002.tif]
